# PET imaging reveals lower kappa opioid receptor availability in alcoholics but no effect of age

**DOI:** 10.1038/s41386-018-0199-1

**Published:** 2018-09-06

**Authors:** Aishwarya Vijay, Dana Cavallo, Alissa Goldberg, Bart de Laat, Nabeel Nabulsi, Yiyun Huang, Suchitra Krishnan-Sarin, Evan D. Morris

**Affiliations:** 1 Department of Radiology and Biomedical Imaging, Yale University, New Haven, CT USA; 2 Department of Psychiatry, Yale University, New Haven, CT USA; 3 Department of Biomedical Engineering, Yale University, New Haven, CT USA

## Abstract

Opioid receptors are implicated in alcoholism, other addictions, withdrawal, and depression, and are considered potential pharmacological targets for treatment. Our goal in the present study was to compare the availability of kappa opioid receptors (KOR) between an alcohol-dependent cohort (AD) and a healthy control cohort (HC). Sixty-four participants—36 AD and 28 HC—underwent PET scans with [^11^C]LY2795050, a selective kappa antagonist tracer. Partial-volume correction was applied to all PET data to correct for atrophy. Volume of distribution (*V*_T_) of the tracer was estimated regionally as a measure of KOR availability. *V*_T_ values of AD versus HC were compared for 15 defined ROIs. Multivariate analysis showed a main effect of group on *V*_T_ across these 15 ROIs. Post hoc tests showed that AD had significantly lower *V*_T_ and thus a lower KOR availability than HC in amygdala and pallidum (corrected for multiple comparisons). Exploratory analysis of change in *V*_T_ with age was conducted; *V*_T_ was not found to vary significantly with age in any region. Our findings of lower *V*_T_ in AD versus HC in multiple regions are in contrast to findings in the mu and delta opioid receptor systems of higher *V*_T_ in AD versus HC. Although age-related decline in receptors has previously been observed in the mu opioid receptor system, we found that KOR availability does not change with age.

## Introduction

Alcohol use disorders (AUDs) affect over 15 million men and women nationwide [1]. The success rate for treatment of alcohol addiction varies widely depending on both individual genetics and the treatment modality used [2, 3]. Therefore, there is a considerable need for new approaches to treating AUDs. Understanding neurobiological mechanisms that underlie alcohol drinking will be important in defining novel targets for AUD treatment.

Alcohol increases endogenous opioid transmission, which is partly responsible for its reinforcing effects [4–6]. The endogenous opioid system is made up of three groups of receptors—the mu (MOR), delta (DOR), and kappa opioid receptors (KOR) [7]. There is a large body of evidence that all three systems are involved in initiating and maintaining excessive alcohol consumption, leading to AUDs—see Reed et al. for review [8].

Approved treatment for AUDs, such as the opioid antagonist naltrexone, have non-selective effects on the opioidergic system. Understanding baseline differences in the status of opioid receptor systems following alcohol drinking, such as upregulation or downregulation of MOR, DOR, and KOR as a consequence of alcoholism, may suggest treatment strategies based on an individual’s opioid receptor profile. Using in vivo PET brain imaging with selective radio ligands to assess and quantify differences in opioid receptor profiles could aid in the development of such new strategies to target effects of alcohol and alleviate alcohol addiction [9].

Variability in clinical response to opioid-targeted treatments, such as naltrexone, has been linked to sex, family history of alcoholism and alcohol craving. The evidence of sex differences in naltrexone response is largely inconclusive. While some clinical trials concluded that men responded better to naltrexone [10, 11], and that women did not seem to respond to naltrexone [12], others found no differences [13], including the large multisite COMBINE trial [14]. Krishnan-Sarin et al. demonstrated that in male alcoholics, administration of 100 mg of naltrexone reduced drinking in the males with a family history of alcoholism but increased drinking in the family history-negative cohort [15]. Some other clinical predictors of naltrexone treatment include having a high level of alcohol craving, high level of stimulation during drinking, or a strong family history of alcoholism [16, 17]. Examining receptor differences could aid in understanding the molecular mechanisms behind these diverging clinical responses to opioid-targeted treatment.

### Sex differences in receptor levels

A growing body of research suggests that sex modulates the pharmacological effects of opiate medications as well as endogenous opioids [18]. We previously observed that healthy males have a higher level of available KOR than healthy females [19]. Zubieta et al. reported the opposite for MOR. That is, greater MOR receptor availability was found in women compared to men using PET and [^11^C]carfentanil [20]. In a recent review, Chartoff et al. reported that in multiple rat studies, KOR upregulation potentiates reward-related effects of drug abuse in males only [21]. These findings may explain the clinical studies that have shown higher efficacy of naltrexone in reducing drinking days and drinks/day in males compared to females [22, 23].

### Mu opioid receptor system

MOR plays a role in alcohol reinforcement and dependence [24]. The MOR system, in particular, has been shown to work in apparent opposition to KOR in both pre-clinical and clinical models. Binding of β-endorphin to MOR induces a positive mood state whereas binding of dynorphin to KOR induces a negative mood state and negative symptoms [25, 26].

Quantification of MOR has been studied, both at the pre-clinical and clinical levels. In alcohol-addicted rats, selective blockade of MOR was sufficient to suppress alcohol drinking [27]. Clinical data, using [^11^C]carfentanil (a PET ligand that selectively binds to MOR), indicate that alcohol-dependent participants have upregulated MOR compared to healthy controls in multiple brain regions as well as higher self-reported craving after a 1–3 week period of abstinence [28, 29]. Conversely, Bencherif et al. found a lower mean MOR-binding potential (BP_ND,_ a measure of available receptors) in alcoholics compared to healthy controls [30]. Thus, the results for MOR levels in alcoholics versus healthy controls remain mixed.

### Kappa opioid receptor system

The binding of dynorphin to KOR, which is generally considered to be associated with aversive feelings, results in decreased dopamine transmission in the brain’s reward pathway [31]. Data suggest that the absence of KOR in KOR-knockout mice results in greater drinking and increased dopamine release in response to alcohol [32]. In rats, chronic alcohol exposure causes downregulation of KOR [33, 34]. This may be a case of positive feedback, since lower levels of KOR may reduce the negative effects of drinking. This finding could apply to humans—alcoholics may have down-regulated KOR compared to healthy controls. If true, treatments intended for patients with AUDs that target KOR may have reduced efficacy as these patients have reduced levels of the target receptor available at baseline.

Only a few other papers report on work that has been done quantifying KOR, in humans, in vivo. The majority of this has been done in controls [35–37] despite the likelihood that the receptors play an important role in alcoholism—see Bruijnzeel et al. for review [38]. Measuring KOR in vivo with PET requires a tracer that is selective for the desired target. Such a tracer was not previously available. Thanks to the recent development of a selective kappa antagonist [39] and its PET tracer analog [^11^C]LY2795050 [40, 41], it is now possible to safely and reproducibly probe the KOR system in human participants, in vivo [19]. Studies have shown that binding of the tracer is reproducible and reliable in regions with moderate and high KOR density in both humans and primates, and [^11^C]LY2795050 has been used to show baseline sex differences in KOR density [19, 36]. The present study aims to explore the differences in KOR availability regionally, in the brain, between alcohol-dependent subjects (AD) and matched healthy control subjects (HC).

## Materials and methods

### Participants

Thirty-six AD (25 male, 11 female) and 28 HC (19 male, 9 female) participants between 19 and 59 years of age were recruited via posted and online advertisement. AD (35.4 ± 9.4 yrs.) and HC (33.5 ± 11.3 yrs.) participants were well matched for age (*p* = 0.74). Demographic characteristics are shown in Table 1.

**Table 1:** Demographics for AD and HC subjects

Characteristics	AD (*n* = 36)	HC (*n* = 28)
Mean years of age (SD)	35.4 (9.4)	33.5 (11.3)
Gender (*n*)		
Male	25	19
Female	11	9
Race (*n*)		
Caucasian	17	15
Black	19	13
Smoking status (*n*)^a^		
Non-smokers	18	24
Smokers	18	4
Family history of alcoholism (*n*)^b^		
FHP	20	
FHN	16	
Smoking measures: mean (SD)^b^		
Peak number of cigarettes/day	11.9 (7.1)	
Years of cigarette use	17.5 (10.6)	
Fagerstrom nicotine dependence score^c^	4.1 (2.3)	
Drinking measures: mean (SD)^b^		
Age at which criteria for alcohol dependence were met	28.1 (8.7)	
Years of dependent alcohol drinking	6.8 (6.6)	
ADS score	10.3 (5.3)	
Number of drinks/drinking day in past 30 days	8.2 (3.2)	
Number of drinking days/week in past 30 days	6.0 (0.9)	
Pre-enrollment Yale Craving Scale	3.9 (6.4)	
Pre-enrollment CIWA-Ar	.08 (.03)	
Pre-enrollment OCDS	11.8 (5.3)	

*AD* alcohol-dependent subjects, *HC* healthy control subjects, *FHP* family history positive, *FHN* family history negative, *ADS* Alcohol Dependence Scale, *CIWA* Clinical Institute Withdrawal Assessment, *OCDS* Obsessive Compulsive Drinking Scale

^a^The majority of smokers were in the AD cohort

^b^Not taken for the healthy control subjects

^c^Heatherton et al. [70]

^d^Sullivan et al. [44]

### Subject inclusion and exclusion criteria

The AD subjects as well as a small fraction of the HC participants (4/28) were participants in a study that administered naltrexone to subjects over the course of a week, after a baseline scan (i.e., a scan prior to administration of naltrexone). The majority of the HC participants (*n* = 24) were part of a study in which subjects received a single dose of naltrexone, immediately following a baseline scan. For the purposes of the present work, only data from the baseline scans of both studies were used, and all subjects were in an un-medicated state. The Yale IRB approved all procedures for these studies and written informed consent was obtained from all subjects (HIC# 1011007710, HIC# 1102008008).

All AD participants received an initial interview, a physical examination and a psychological evaluation from a licensed clinical psychologist. For inclusion in the study, AD participants had to meet current DSM-IV criteria for alcohol dependence at the time of intake and had to be actively drinking at or above NIAAA-defined hazardous levels (≥20 drinks/week for women; >25 drinks/week for men) as determined by completion of a 90-day Time Line Follow Back (TLFB) assessment [42]. HC participants did not drink more than the NIAAA-recommended guidelines < 10 drinks/week) and had never met DSM-IV criteria for either alcohol abuse or dependence in their lifetime. In addition, AD participants were excluded if they had a CIWA-Ar withdrawal score greater than 8 [43]. CIWA-Ar scores, determined for AD participants only, were universally low with very low variability across participants. Other exclusion criteria for HC have been described previously [19]. Participants in the study that administered naltrexone over a week (36 AD; 4 HC) were considered to be smokers if they were current daily smokers (≥1 cigarette/day). For the study that administered a single dose of naltrexone (24 HC), smoking was an exclusion.

Immediately prior to PET procedures, urine toxicology screens and breath alcohol tests were conducted in all participants to verify abstinence from alcohol and drugs. All female subjects of child-bearing potential were required to take a urine pregnancy test and were excluded from the study if the test was positive. For menstruating female participants, the timing of PET scanning within the menstrual cycle was fixed so that females were studied (whenever possible) during the follicular phase of their menstrual cycle.

### Imaging

[^11^C]LY2795050, a selective kappa antagonist tracer [40], was synthesized as reported previously [41]. As shown in Table 2, neither the injected dose of radioactivity, nor the injected mass were different for AD and HC groups.

**Table 2:** Injection parameters

[^11^C]LY2795050	AD (mean ± SD)	HC (mean ± SD)	*p*
Number	36 (56.3%)	28 (43.7%)	
Injected activity/BW ^a^	0.15 ± 0.07	0.13 ± 0.06	0.68
Molar activity^b^	0.65 ± 0.36	0.57 ± 0.31	0.45
Mass dose/body weight^c^	0.10 ± 0.03	0.09 ± 0.05	0.83

^a^In mCi/kg

^b^In mCi/nmol at time of injection

^c^In µg/kg

Prior to their PET scan, participants underwent magnetic resonance imaging (MRI) on a 3T whole-body scanner (Trio, Siemens Medical Systems, Erlangen Germany). A structural T1 MRI was acquired for anatomical localization of the PET brain regions of interest. MR co-registration scans were 18 min long and occurred on a separate day from the PET scan. PET imaging occurred only if individuals had a negative breath alcohol test shortly before the start of the PET scan. All PET scans were 90 min long.

PET scans were acquired on a HRRT scanner (Siemens/CTI, Knoxville, TN, USA). [^11^C]LY2795050 was injected as a bolus over 1 min. Dynamic scan data were acquired in list-mode and reconstructed (FWHM resolution ≈ 3 mm) in 27 frames (6×0.5 min, 3×1 min, 2×2 min, 16×5 min) with correction for attenuation, normalization, scatter, randoms and dead time using the iterative MOLAR algorithm [44]. Motion correction at the event-level [45] (Polaris Vicra Tracking System, Northern Digital, Waterloo, Canada) was applied. The free fraction of [^11^C]LY2795050 in plasma (*f*_p_) was found to be ~1%, as previously described [41].

### Image pre-processing

Partial-volume correction (PVC) was performed in native PET space and applied to all PET data from all participants [46]. Estimation of white matter (WM) signal was performed as previously described by Vijay et al. following the method of Giovachini et al. [19]. PVC data was used for both the voxel level and the ROI-based analyses.

Fifteen regions of interest (ROIs) included in this study were taken from the Automated Anatomical Labeling (AAL) template in MNI space [47]. The ROIs were the amygdala, centrum semiovale, caudate, cerebellum, anterior and posterior cingulate cortex, frontal cortex, hippocampus, insula, occipital cortex, ventral pallidum, parietal cortex, putamen, temporal cortex, and thalamus. A mask of the overlapping sub-regions of the caudate and putamen (ventral striatum, dorsal caudate, and dorsal putamen) was applied to all PET data in template space [48]. These regions were examined as they are of particular interest in addiction [49–51]. To determine the correct transformation for each subject from PET to MNI, the summed (0 to 10 min after injection) PET image was first co-registered to the subject’s own high-resolution T1-weighted MR image which was subsequently co-registered to the template [52].

Time activity curves (TACs) were extracted from each of the 15 ROIs using the mean radioactivity in the ROI for each time frame. TACs were fitted with the multi-linear analysis-1 (MA1) model [53] using a metabolite-corrected arterial input function to estimate volume of distribution (*V*_T_) by region.

### Volume of distribution

The primary output measure in this study was volume of distribution (*V*_T_), a normalized measure of steady state tracer uptake in the target tissue.

1 $$\left. {V_{\mathrm{T}}{\mathrm{ = }}\frac{\mathrm{[Tissue]}}{\mathrm{[Plasma]}}} \right|_{{\mathrm {steady}}\,{\mathrm{state}}}$$

[Tissue] is the concentration of tracer in the tissue and [Plasma] is the concentration of tracer in the plasma.

The components of the signal in Eq. 1 are the specific (*V*_S_) and the non-displaceable (*V*_ND_) volumes of distribution, respectively [54].

2 $$V_{\mathrm{T}}{\mathrm{ = }}V_{\mathrm{s}} + V_{\mathrm{ND}}$$

In order to attribute observed differences in *V*_T_ to differences in receptor availability with confidence, *V*_ND_ must not differ between cohorts. *V*_ND_ was calculated using a Lassen plot as previously described [19], taking advantage of the fact that most subjects in both groups were scanned an additional time after pretreatment with naltrexone.

### Regional analysis

A multivariate analysis was performed for the AD versus HC comparison using sex, smoking, and age as separate fixed factors. This was performed for all 15 ROIs at once. An overall main effect of drinking was examined using multivariate analysis, and post hoc *t*-tests between AD and HC were performed across the 15 defined ROIs to confirm significant differences (*p* < 0.05). The reported *p*-values for these post hoc *t*-tests were not corrected for multiple testing, but were compared to the Benjamini–Hochberg threshold [55]. Post hoc *t*-tests between AD and HC were performed for the total cohort (*n* = 64), as well as for male (*n* = 44) and female (*n* = 20) cohorts, separately. The AD cohort was also examined for *V*_T_ differences by ROI-based on smoking status and family history of alcoholism. The HC cohort was not examined for smoking status as only four of the HCs were smokers. Multivariate analysis and univariate analyses examining the effect of age on *V*_T_ were also performed.

### Creation of parametric *V*_T_ maps

Voxel-by-voxel (parametric) maps of *V*_T_ were created from PET data following PVC. As stated above, only baseline scans were used. The MA1 model (t* = 40) was fitted at each voxel in order to calculate parametric *V*_T_ maps. Maps were spatially normalized to MNI space.

Because of noise in the dynamic PET data at the voxel level, the MA1 model occasionally produced poor fits and non-physiological *V*_T_ values at individual voxels. To correct for this, parametric maps of the weighted sum of squared residuals (WSSR) from the MA1 fits were calculated for each subject. The WSSR maps were thresholded at 3 standard deviations above the mean WSSR to identify voxels containing outlier *V*_T_ values. These *V*_T_ values were excluded. To avoid parametric maps with undefined voxels, the excluded *V*_T_ values were replaced with the median *V*_T_ of the 26 nearest-neighbor voxels. Parametric maps for all subjects in the AD cohort alone or HC cohort alone were averaged to create mean *V*_T_ maps [19].

### Voxel-based analyses

Whole-brain analyses were performed in SPM12 (Wellcome Trust Centre for Neuroimaging, London, UK, http://www.fil.ion.ucl.ac.uk/spm/). The processed *V*_T_ maps were smoothed with a 7 mm FWHM Gaussian kernel and masked by a binary gray-matter mask. Parametric statistical models were assumed at each voxel using the general linear model. Voxel-wise two-sample *t*-tests were performed to examine the differences in *V*_T_ between cohorts and voxel-wise *t*-score maps were produced. A primary threshold of voxel-wise *p* < 0.001 and a secondary cluster-extent based threshold of 30 was applied to the t-score maps.

## Results

### *V*_ND_

Non-displaceable volume of distribution, *V*_ND_, was not different between AD and HC groups (*f*(63) = 3.41, *p* = 0.55). Similarly, this was true for males alone (*f*(43) = 2.68, *p* = 0.29) and for females alone (*f*(19) = 1.94, *p* = 0.23).

### Effect of drinking

#### Average parametric images

**Fig. 1:**
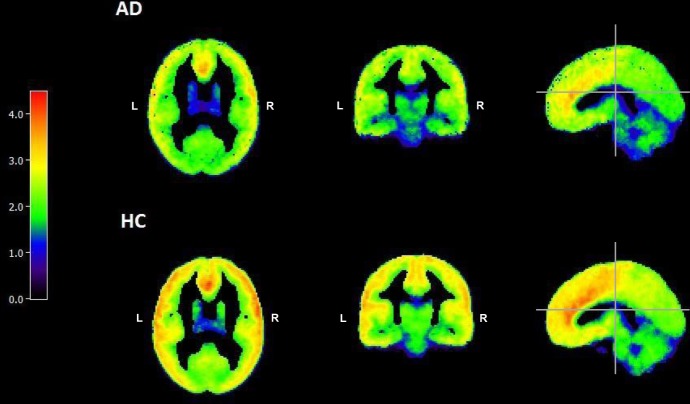
Average parametric *V*_T_ images of AD (*n* = 36) and HC (*n* = 28). *V*_T_ at each voxel was calculated using MA1 (t* = 40). Values outside of 3 standard deviations above the mean WSSR were excluded. Color bar depicts [^11^C]LY2795050 *V*_T_ from 0 (black) to 5.00 (red)

Figure 1 shows the mean *V*_T_ images of AD (*n* = 36) and HC subjects (*n* = 28) after PVC. All subjects in each cohort were used to create the mean images. Images are in MNI space and appear smoothed because they are averages. The mean images are all displayed on the same color scale. Lower *V*_T_ in AD as compared to HC is apparent, visually, in the frontal and parietal cortices.

#### Regional analysis

There was a significant overall effect of drinking status—[^11^C] LY2795050 *V*_T_ was lower in AD vs HC across all regions (*f*(63) = 2.25, *p* < 0.01). Post hoc analysis revealed this effect was driven by group differences in eight regions. There were no significant interactions between drinking status with smoking, sex, or age that would affect *V*_T_ comparisons. There was a significant interaction between age and smoking as measured by a chi-squared test (*χ*^2^ = 3.07, *p* = 0.02)—the likelihood of a subject being a smoker (defined as ≥ 1 cigarette/day) was greater with greater age.

**Fig. 2:**
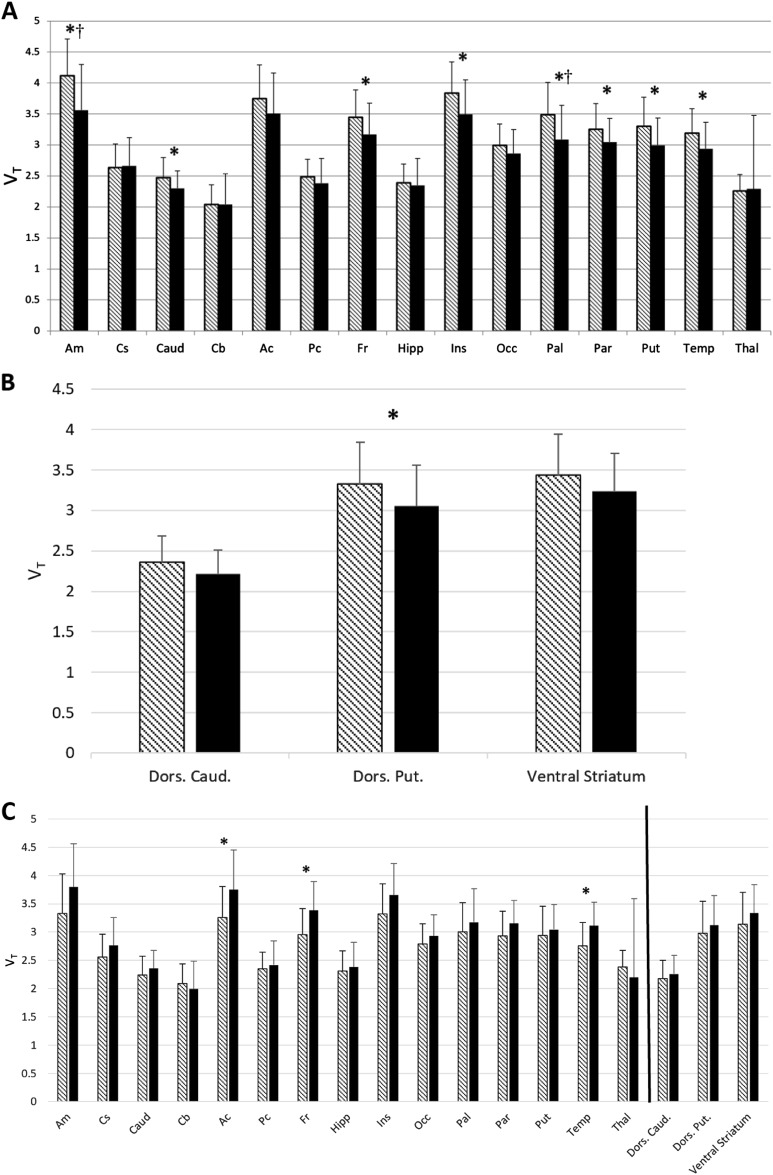
**a**–**c** ROI Analysis of *V*_T_. **a** [^11^C] LY2795050 *V*_T_ in the whole cohort (*n* = 64) by region and drinking status. Multivariate analysis showed a significant main effect for drinking status, i.e., the AD cohort (*n* = 36, solid bars) had lower *V*_T_ than the HC cohort (*n* = 28, hatched bars). Bars represent the mean ± SD of [^11^C]LY2795050 *V*_T_ respectively for amygdala (Am), centrum semiovale (Cs), caudate (Caud), cerebellum (Cb), anterior cingulate cortex (Ac), posterior cingulate cortex (Pc), frontal cortex (Fr), hippocampus (Hipp), insula (Ins), occipital cortex (Occ), ventral pallidum (Pal), parietal cortex (Par), putamen (Put), temporal cortex (Temp), thalamus (Thal). Regions are non-overlapping. *indicates the significant difference between the regional *V*_T_ of AD versus HC, *p* < 0.05 uncorrected for multiple comparisons. † indicates significance after multiple comparison correction. **b** [^11^C] LY2795050 *V*_T_ in the whole cohort (*n* = 64) of alcohol-dependent (AD) (*n* = 36) versus healthy control (HC) subjects (*n* = 28) at the region of interest (ROI) level. Hatched bars represent the HC cohort and solid bars represent the AD cohort. Bars represent the mean ± SD of [^11^C]LY2795050 *V*_T_ respectively for dorsal caudate (Dors. Caud.), dorsal putamen (Dors. Put), and ventral striatum. *indicates the significant difference between the regional *V*_T_ of AD versus HC, *p* < 0.05 uncorrected for multiple comparisons. **c** [^11^C] LY2795050 *V*_T_ in the cohort (*n* = 36) of AD non-smokers (*n* = 18) versus AD smokers (*n* = 18) at the region of interest (ROI) level. Hatched bars represent the non-smokers and solid bars represent the smokers. Bars represent the mean ± SD of [^11^C]LY2795050 *V*_T_ respectively for regions listed in Fig. 2a, b above. The black line separates the striatal sub-regions from the main ROIs. *indicates the significant difference between the regional *V*_T_ of smokers versus non-smokers, *p* < 0.05 uncorrected for multiple comparisons

At the region level (Fig. 2a), *V*_T_ was significantly lower in AD versus HC in eight ROIs. These regions were: amygdala (*f*(63) = 10.3, *p* = 0.002), caudate (*f*(63) = 5.09, *p* = 0.028), frontal cortex (*f*(63) = 5.84, *p* = 0.019), insula (*f*(63) = 7.38, *p* = 0.009), pallidum (*f*(63) = 10.11, *p* = 0.002), parietal cortex (*f*(63) = 4.55, *p* = 0.037), putamen (*f*(63) = 7.42, *p* = 0.008), and temporal cortex (*f*(63) = 6.33, *p* = 0.014). When corrected for multiple testing with the Benjamini–Hochberg procedure, significant differences were still found in the amygdala (*t*(62) = 3.36, *p* = 0.002) and pallidum (*t*(60) = 2.96, *p* = 0.005). Of the striatal sub-regions of specific interest for drug abuse, *V*_T_ was significantly lower in AD versus HC in the dorsal putamen (*f*(63) = 4.85, *p* < 0.05 uncorrected), and all three sub-regions showed lower *V*_T_ in AD compared to HC (Fig. 2b). To verify that observed group differences were not an artifact of partial-volume correction, we analyzed our data without PVC and obtained essentially the same differences (see Supplementary Fig. 1).

### Effect of sex

The AD versus HC comparison was also examined by sex. In males (25 AD, 19 HC), 9 regions were significantly lower in AD than HC (*f*(43) = 3.68, *p* < 0.05, uncorrected). The female cohort (9 AD, 11 HC) was underpowered when compared to males, and thus, only 1 region—the amygdala—was significantly lower (*f*(19) = 1.22, *p* < 0.05, uncorrected) in AD than HC. Although only the amygdala reached significance, all regions in the female cohort except for cerebellum and hippocampus had a lower mean *V*_T_ in AD compared to HC. These analyses are exploratory, as neither a main effect of sex nor an interaction between AD and sex were found to be significant in the multivariate analyses. These results are shown in Supplementary Fig. 2.

### Effect of smoking

Regional analyses were performed in the AD cohort alone, comparing AD smokers (*n* = 13) versus AD non-smokers (*n* = 23). AD smokers had a significantly higher *V*_T_ versus AD non-smokers in the anterior cingulate cortex (*f*(35) = 2.93), frontal cortex (*f*(35) = 5.84), and temporal cortex (*f*(35) = 6.31) (all regions *p* < 0.05, uncorrected) (Fig. 2c). All regions with the exception of the cerebellum and thalamus showed the same pattern of higher *V*_T_ in AD smokers versus AD non-smokers. Regional analyses were also performed in non-smokers alone, comparing the AD and HC cohorts. HC non-smokers had significantly higher *V*_T_ versus AD non-smokers in fourteen regions, including striatal sub-regions (Supplementary Fig. 2). As there was no overall effect of smoking, these analyses must be considered exploratory.

### Effect of family history

Regional analyses were performed in the AD cohort alone, comparing *V*_T_ in subjects with a family history of alcoholism to (*n* = 19) versus those with no family history (*n* = 17). There were no regions of significant difference and no pattern or trend in differences (i.e., family history positive subjects had a non-significantly higher *V*_T_ in most/all regions or vice versa).

### Effect of age

**Fig. 3:**
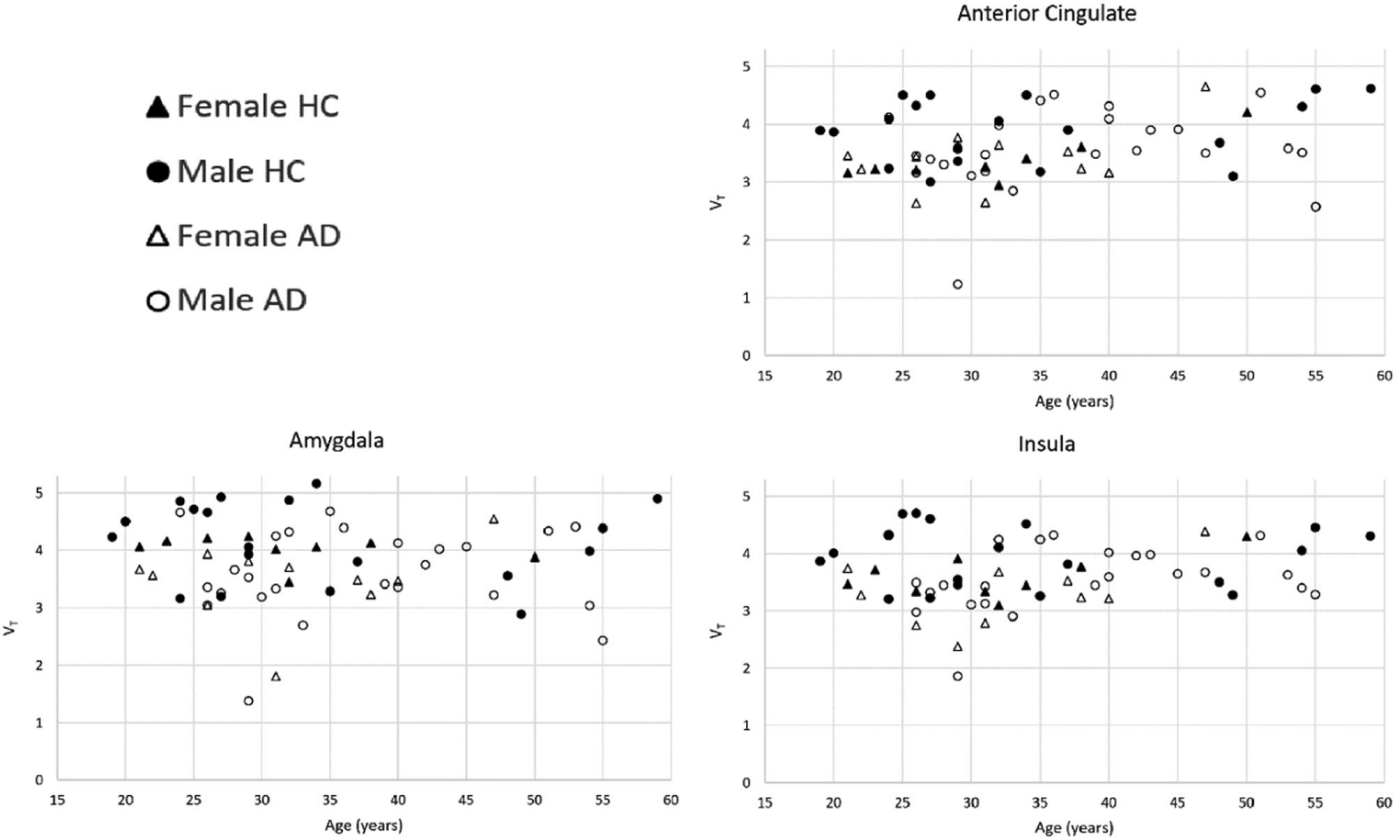
*V*_T_ versus age in the three highest binding regions. [^11^C]LY2795050 V_T_ versus age, separated by both cohort (AD versus HC) and sex (male versus female)

Multivariate analyses showed that there was no significant effect of age on *V*_T_ (*f*(63) = 1.05, *p* = 0.54, uncorrected). Plots of *V*_T_ versus age by sex and drinking status for the three highest binding regions are shown in Fig. 3. Additionally, HC subjects had higher *V*_T_ interpolated back to birth as compared to AD in every region but the thalamus (Supplementary Fig. 3).

### Voxel-based analysis

**Fig. 4:**
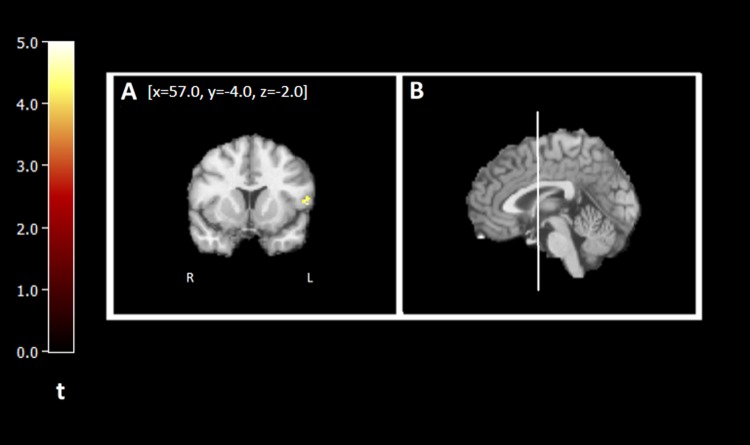
Voxel-by-voxel analysis of *V*_T_ in AD versus HC. [^11^C]LY2795050 *V*_T_ images from a two-sample *t*-test between AD and HC using statistical parametric mapping (SPM) overlayed with an atlas. Regions with a significant difference (*p* < 0.001, family-wise error [FWE]) between AD and HC, where HC > AD, are outlined as follows: **a** frontal cortex, and **b** Location of the coronal slice shown in the sagittal view of the brain—white line indicates location of coronal slice A in sagittal plane. **a** Coordinates are (*x* = 57.0, *y* = −4.0, *z* = −2.0). Colored legend depicts *t*-scores ranging from 0 to 5

SPM analysis of [^11^C]LY2795050 *V*_T_ in AD and HC confirmed that AD had lower *V*_T_ in multiple brain areas. Voxel-wise results are shown in Fig. 4. Group differences were seen in two main regions: frontal cortex and temporal cortex. Both of these regions were also found to be significantly different in the ROI analysis.

## Discussion

To our knowledge, this is the first study on the difference of available KOR levels in the brains of AD versus HC human subjects, in vivo. In our PET study with a selective KOR antagonist tracer, we found that alcohol-dependent subjects (AD) have a lower *V*_T_ for the [^11^C]LY2795050 tracer compared to healthy controls (HC) across all regions of the brain. We also found that non-displaceable volume of distribution (*V*_ND_) was not different between AD and HC. Thus, we can interpret our results as demonstrating more available KOR-binding sites in HC than AD subjects. ROI analysis showed a significant difference (uncorrected) in *V*_T_ between AD and HC in eight of fifteen brain regions and one of three striatal sub-regions. Of these eight regions, the amygdala and pallidum were significant after Benjamini–Hochberg multiple testing correction. When the cohort was split by sex, the same regions remained significantly different in the male cohort, although this analysis is exploratory. Although similar trends were observed in the female cohort, we were significantly underpowered for this comparison, a common issue for research on alcoholism and other substance abuse [56]. While there were no overall effects of smoking or age, at a regional level, the slope of *V*_T_ versus age was positive in almost all regions examined. The slope was greater in the AD cohort versus the HC cohort, but this difference was not significant.

Previous research by both Weerts et al. and Heinz et al. on the MOR system demonstrated higher levels of available receptors (BP_ND_—non-displaceable binding potential) in AD versus HC in some regions, such as the amygdala, insula, ventral striatum, putamen, and caudate [28, 29]. Our data preliminarily suggest that the opposite is true in KOR (i.e., AD have a significantly lower *V*_T_ than HC in multiple regions). This contrast in observations lends support to the idea that KOR and MOR systems may work in opposition to each other [25, 26]. Furthermore, our findings are supported by pre-clinical evidence from rats showing that chronic alcohol exposure causes downregulation of KOR [33, 34]. While the clinical implications of these findings remain to be determined, it is possible that the efficacy of treatments for alcohol dependence that target the opioid systems may be related to levels of KOR. Finally, the collective availability of selective tracers for all three opioid receptor subtypes suggests the possibility of comprehensive studies of opioid receptor systems in the same individuals.

Our exploratory findings of lower *V*_T_ for the AD cohort versus HC in the insula, amygdala, frontal cortex and dorsal striatum (caudate and putamen) are particularly interesting, despite only the amygdala surviving the multiple testing correction. They are consistent with an anatomical imaging study that showed a markedly decreased amygdala volume in AD in relation to HC subjects [57]. The insula has been implicated in the process of drug craving, in general [58]. There is evidence that the frontal lobes are particularly vulnerable to damage from alcoholism [50]. Finally, while the role of the ventral striatum in drinking has been reported on extensively [59–61], our findings suggest a potential role of the dorsal striatum that has not previously been reported.

Examination of the effect of age on *V*_T_ in this study was exploratory. Previous literature in KOR and aging is confined to pre-clinical models. Gosnell et al. found a decreased effect of naloxone on feeding in aged rats, suggesting that KOR may decrease or be inhibited with age [62]. Lower activity of dynorphin (the endogenous opioid ligand for KOR) has been found in the hypothalamus in aging rodents [63].

In this study, however, age was not found to have an overall effect on KOR availability. This is in contrast to previous studies that have shown a decline in neuro-receptors in age [64], especially in states of addiction [65]. Studies have shown age-related decreases in the dopaminergic, seretonergic, and glutaminergic receptor systems, as reviewed by Dowling et al. [66]. Rinne et al. and Wang et al. both reported an age-dependent decline in human dopamine D1 receptors in both striatal and extra-striatal regions [67, 68]. Imaging studies of cocaine abusers suggests that the effects of reductions in dopamine receptors with age may be exacerbated by drug abuse [65, 69].

Some limitations need to be considered. BP_ND_ would be a more ideal primary endpoint as it is a more direct metric of receptor availability. Unfortunately, there is no validated reference region for KOR. A blocking study could be used to estimate *V*_ND_ and from there, *V*_S_ (Eq. 2), but blocking with naltrexone was only performed in a subset of the HC cohort. However, *V*_ND_ was compared between AD and HC for all subjects who had a blocking study and was not found to be significantly different. Thus, we are confident that *V*_T_ differences represent differences in specific binding (*V*_S_). The female cohort in this study was under-powered. Thus, findings from the analysis of the female-only cohort did not reach significance, but the trends observed were consistent with those of the male cohort.

We found that alcohol-dependent subjects have a significantly lower KOR availability compared to healthy controls across multiple regions of the brain. *V*_T_ was used as an indirect measure of receptor availability. Although the study was not longitudinal, there was no apparent change in receptor number with age in either the HC or AD cohorts. Nonetheless, our findings point toward a possible biological basis for differences in clinical effectiveness of opioid-targeted medications. Future development of these treatments must consider the KOR system as a target.

## Electronic supplementary material

Supplemental Figure 1

Supplemental Figure 2

Supplemental Figure 3
