# Supplementary material for: PET imaging reveals lower kappa opioid receptor availability in alcoholics but no effect of age
Source: Neuropsychopharmacology. 2018 Sep 6;43(13):2539–47. doi: 10.1038/s41386-018-0199-1 (PMC6224533; doi:10.1038/s41386-018-0199-1)
Supplement: Supplementary file 1 — Supplemental Figure 1 [file 41386_2018_199_MOESM1_ESM.pdf]

Supplemental Figure 1. Comparison of results with and without PVC

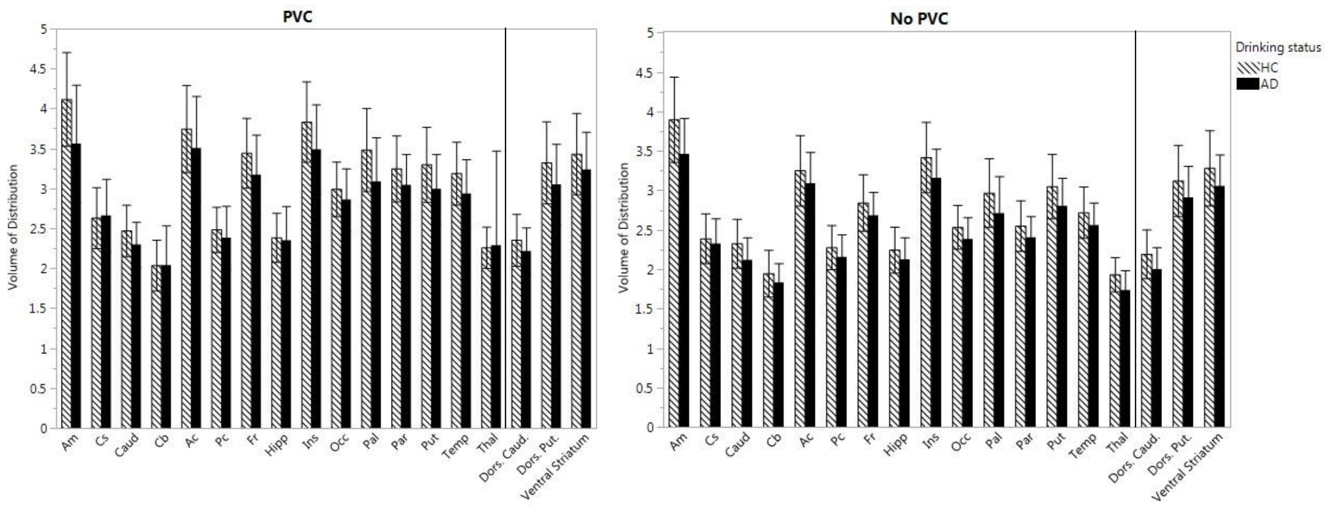

Visual comparison of estimated volumes of distribution (VT) with (Left panel) or without (Right panel) partial volume correction (PVC) for amygdala (Am), centrum semiovale (Cs), caudate (Caud), cerebellum (Cb), anterior cingulate cortex (Ac), posterior cingulate cortex (Pc), frontal cortex (Fr), hippocampus (Hipp), insula (Ins), occipital cortex (Occ), ventral pallidum (Pal), parietal cortex (Par), putamen (Put), temporal cortex (Temp), thalamus (Thal), dorsal caudate (Dors. Caud), dorsal putamen (Dors. Put.), and ventral striatum. The hatched bars represent the HC cohort and the solid bars represent the AD cohort. The black line separates the striatal sub-regions from the main ROIs.
