# Supplementary material for: PET imaging reveals lower kappa opioid receptor availability in alcoholics but no effect of age
Source: Neuropsychopharmacology. 2018 Sep 6;43(13):2539–47. doi: 10.1038/s41386-018-0199-1 (PMC6224533; doi:10.1038/s41386-018-0199-1)
Supplement: Supplementary file 2 — Supplemental Figure 2 [file 41386_2018_199_MOESM2_ESM.pdf]

Supplemental Figure 2 (A-C). ROI Analysis by gender and smoking status

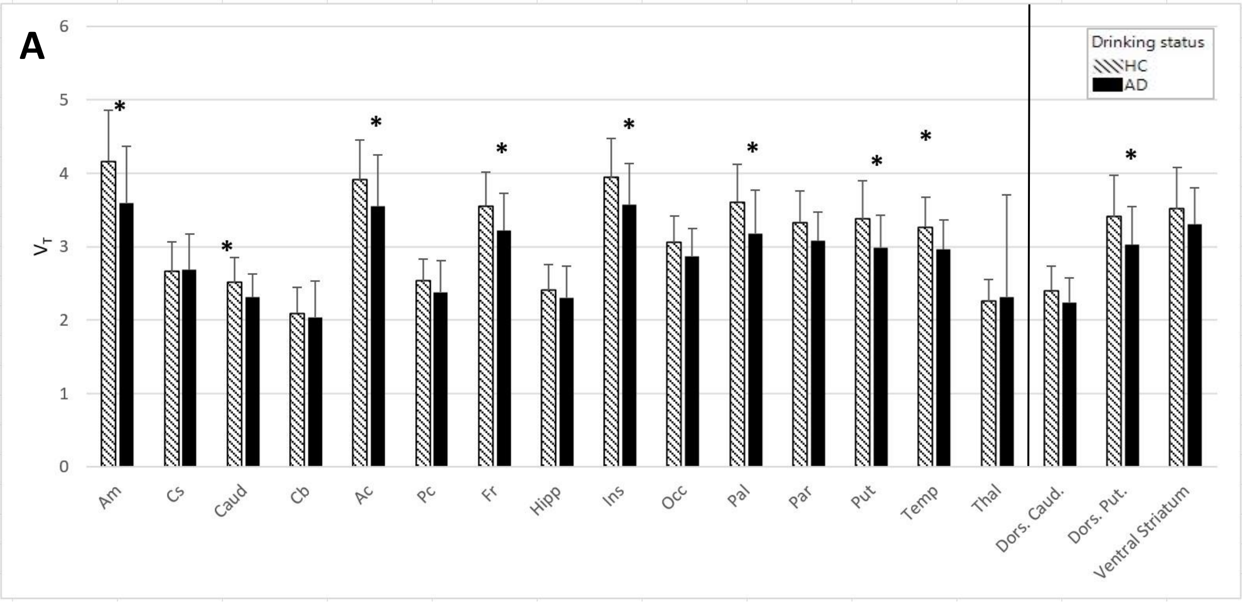

(A) [^{11}C]LY2795050 V_T in a cohort (n=44) of male AD (n=25) versus male HC (n=19) subjects at the region of interest (ROI) level. Hatched bars represent the HC cohort and solid bars represent the AD cohort. Bars represent the mean ± SD of [^{11}C]LY2795050 V_T respectively for amygdala (Am), centrum semiovale (Cs), caudate (Caud), cerebellum (Cb), anterior cingulate cortex (Ac), posterior cingulate cortex (Pc), frontal cortex (Fr), hippocampus (Hipp), insula (Ins), occipital cortex (Occ), ventral pallidum (Pal), parietal cortex (Par), putamen (Put), temporal cortex (Temp), thalamus (Thal), dorsal caudate (Dors. Caud), dorsal putamen (Dors. Put.), and ventral striatum. The black line separates the striatal sub-regions from the main ROIs. * = $p<0.05$, uncorrected for multiple comparisons.

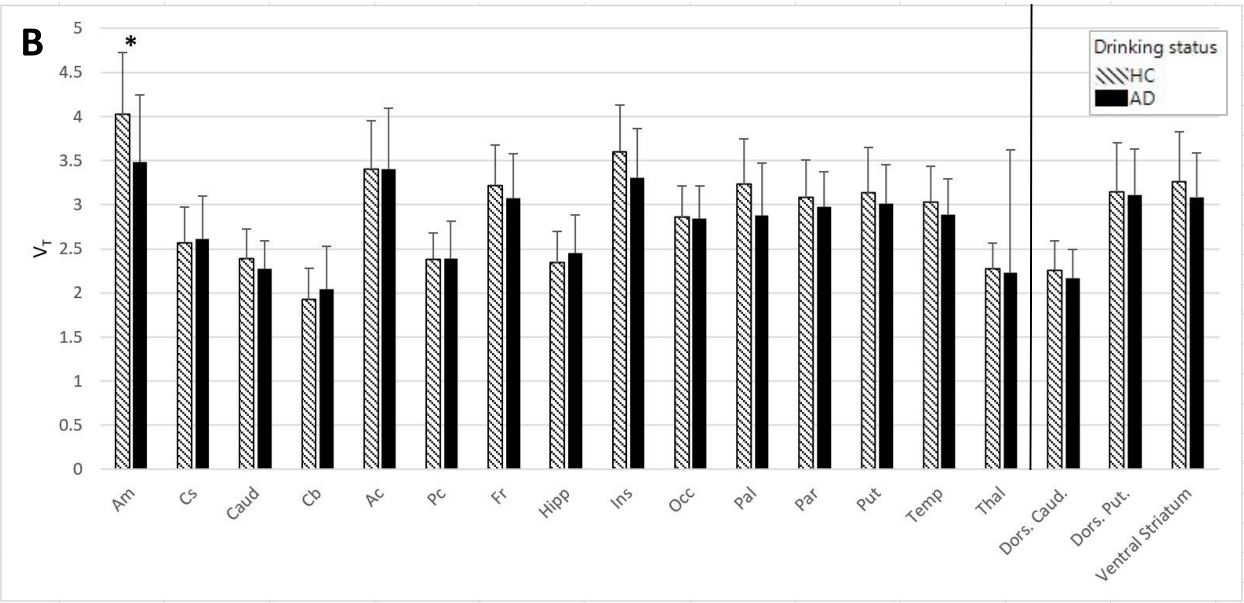

(B) [^{11}C] LY2795050 V_T in a cohort (n=20) of female AD (n=11) versus female HC (n=9) subjects at the region of interest (ROI) level. Hatched bars represent the HC cohort and solid bars represent the AD cohort. Bars represent the mean ± SD of [^{11}C]LY2795050 V_T respectively for regions listed in panel A above. The black line separates the striatal sub-regions from the main ROIs. * = $p<0.05$, uncorrected for multiple comparisons.

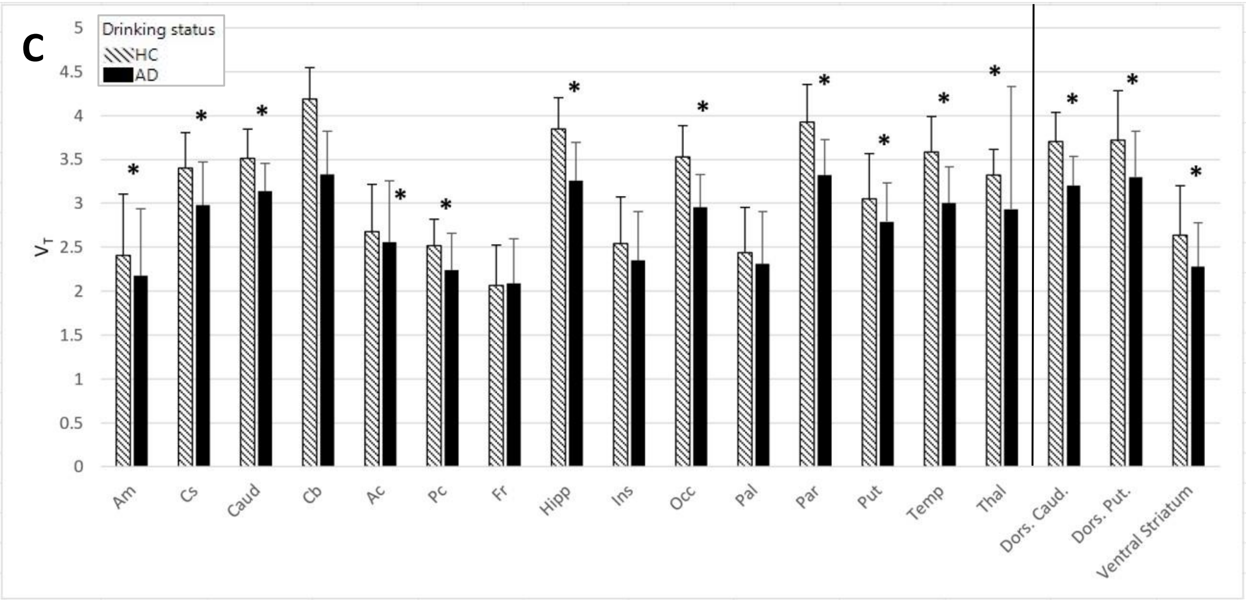

(C) [^{11}C] LY2795050 V_T in the cohort (n=44) of AD non-smokers (n=18) versus HC non-smokers (n=24) at the region of interest (ROI) level. The hatched bars represent the HC cohort and the solid bars represent the AD cohort. Bars represent the mean ± SD of [^{11}C]LY2795050 V_T respectively for regions listed in panel A. The black line separates the striatal sub-regions from the main ROIs. * = $p<0.05$, uncorrected for multiple comparisons.
