# Supplementary material for: PET imaging reveals lower kappa opioid receptor availability in alcoholics but no effect of age
Source: Neuropsychopharmacology. 2018 Sep 6;43(13):2539–47. doi: 10.1038/s41386-018-0199-1 (PMC6224533; doi:10.1038/s41386-018-0199-1)
Supplement: Supplementary file 3 — Supplemental Figure 3 [file 41386_2018_199_MOESM3_ESM.pdf]

Supplemental Figure 3. KOR Availability at Birth in AD versus HC

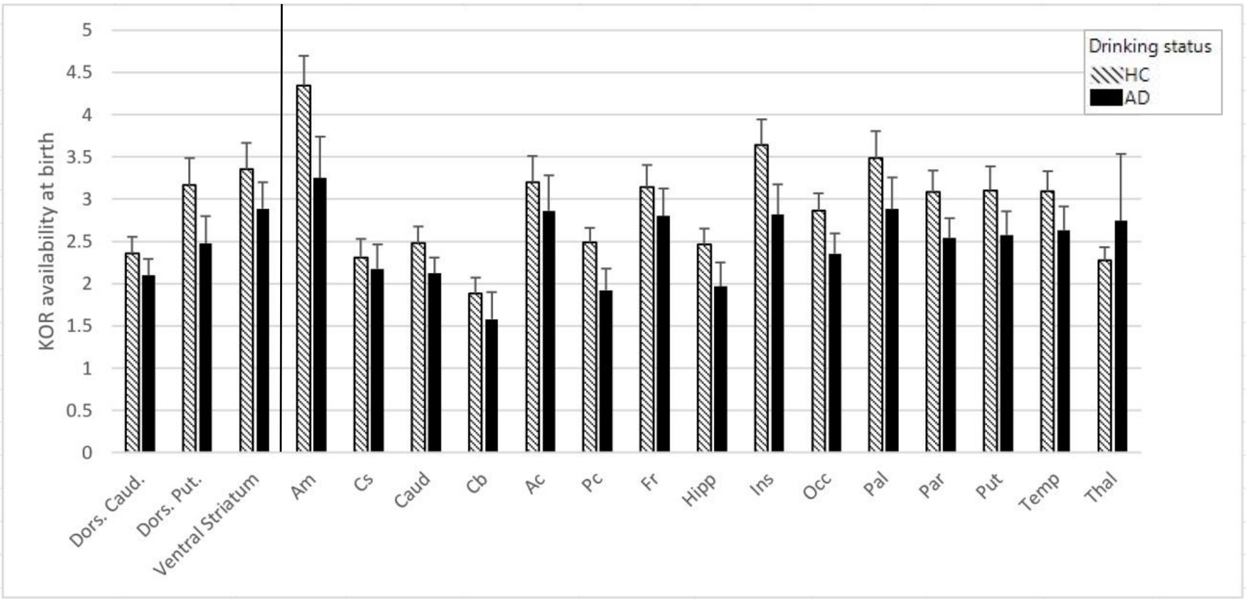

Intercepts of [^{11}C]LY2795050 V_T in function of age in the current sample (n=64) at ROI level. The hatched bars represent the HC cohort and the solid bars represent the AD cohort. If a linear relation could be assumed outside the age range of the current sample, this finding might be indicative of differences in KOR availability at birth in the amygdala (Am), centrum semiovale (Cs), caudate (Caud), cerebellum (Cb), anterior cingulate cortex (Ac), posterior cingulate cortex (Pc), frontal cortex (Fr), hippocampus (Hipp), insula (Ins), occipital cortex (Occ), ventral pallidum (Pal), parietal cortex (Par), putamen (Put), temporal cortex (Temp), thalamus (Thal), dorsal caudate (Dors. Caud), dorsal putamen (Dors. Put.), and ventral striatum. The black line separates the striatal sub-regions from the main ROIs.
